# 562. Tocilizumab Use in the Second Trimester Pregnant Patients with Severe Covid-19 Pneumonia and their Maternal and Fetal Outcomes: Two Case Reports

**DOI:** 10.1093/ofid/ofab466.760

**Published:** 2021-12-04

**Authors:** Fatima iqbal, Shiema A Ahmed, Kamran Mushtaq, Faraj S Howady, Fatima Rustom, Muna Almaslamani

**Affiliations:** 1 Hamad Medical Corporation, Doha, Ad Dawhah, Qatar; 2 CDC- Hamad medical Corporation, Doha, Ad Dawhah, Qatar; 4 Communicable Disease Center, Doha, Ad Dawhah, Qatar

## Abstract

**Background:**

Tocilizumab is an interleukin-6 monoclonal antibody with widespread use in rheumatologic conditions. Observational studies have shown a promising role of Tocilizumab in severe COVID-19 patients with cytokine storm syndrome. Data about tocilizumab use in pregnant patients is limited. We report two outcomes of two pregnant patients with COVID-19 in the second trimester who received tocilizumab

**Methods:**

A 24-year-old 20 weeks pregnant lady with a history of asthma and gestational diabetes mellitus presented with three days history of fever, cough and shortness of breath (Figure 1). She was clinically stable but later developed ARDS and developed increased oxygen demand up to 10 liters/min. She received Tocilizumab on. Patient was observed in a high dependency unit but did not require mechanical ventilation. Patient was discharged home with full recovery and later delivered a healthy baby. Timeline of medicines used during hospital (Figure 2). Case 2: 39-year-old 23 weeks pregnant lady presented with seven days history of fever cough and shortness of breath (Figure 1). On presentation, she had progressive worsening hypoxic respiratory failure and was intubated. Patient had her nasopharyngeal swab for CODI-19 RT PCR was positive. The patient had severe ARDS requiring ECMO (extracorporeal membrane oxygenation) for respiratory support. Tocilizumab 400 mg was given on the presentation, along with other medications (Figure 3). Patient had regular monitoring of fetus; however, she had intrauterine fetal demise on day 14. Patient It is unclear if IUFD was due to using of tocilizumab or severity of COVID19 itself. The patient stayed in ICU for 20 days and was discharged after full recovery.

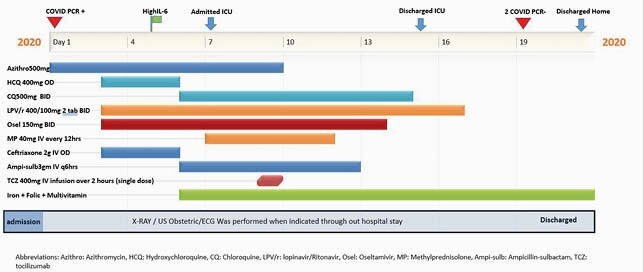

Figure 1. Case 1 treatment timeline. Abberviations: Azithro: Azithromycin, HCQ: Hydroxychloroquine, CQ: Chloroquine, LPV/r: lopinavir/Ritonavir, Osel: Oseltamivir, MP: Methylprednisolone, Ampi-sulb: Ampicillin-sulbactam, TCZ: tocilizumab

Figure 2. Case 2 treatment timeline

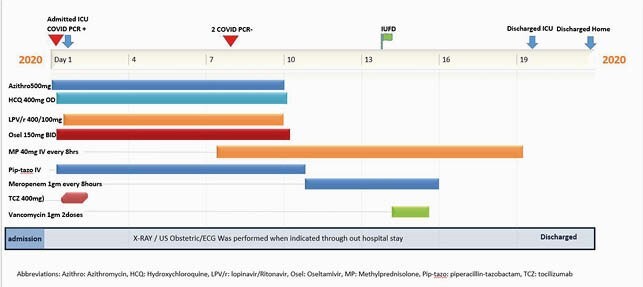

**Results:**

Learning points: Tocilizumab use in pregnant patients with severe COVID-19 pneumonia during the second trimester improved maternal outcomes in our cases. Tocilizumab use may be associated with worse fetal outcomes, including intrauterine fetal demise (IUFD).

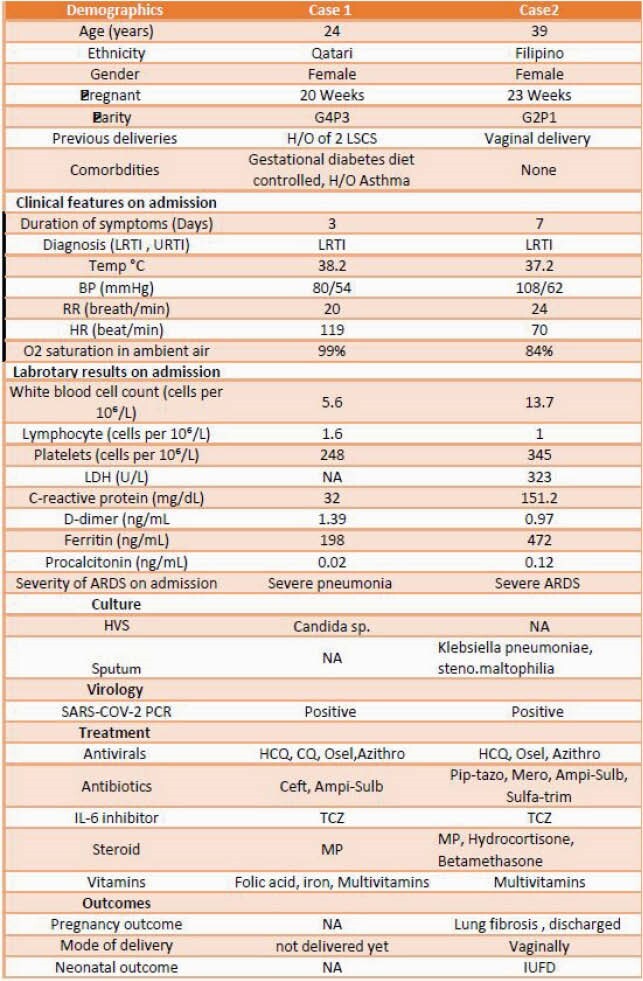

Figure 3. Table of clinical characteristics, pregnant outcomes. Abbreviations: LRTI: lower respiratory tract infection, HCQ: Hydroxychloroquine, CQ: chloroquine, Osel: Oseltamivir, Cef: Ceftrixone, Ampi-Sulb: ampicillin-sulbactam, Azithro: Azithromycin, TCZ: tocilizumab, MP: methylpredinisolone, H/O: History of, LSCS: C-section, NA: not available. Pip-tazo: Piperacillin-tazobactam, Mero: Meropenem, Sulfa-trim: Sulfamethoxazole-Trimethoprim, IUFD: Intrauterine fetal death.

**Conclusion:**

The pharmacological management of pregnant patients with severe COVID-19 pneumonia poses significant challenges. The use of Tocilizumab may improve maternal outcomes but may also increase the risk of worse fetal outcomes. Caution should be exercised in using this agent, and risks and benefits should be discussed with the patients.

**Disclosures:**

**All Authors**: No reported disclosures

